# Posttraumatic Cholesteatoma Complicated by a Facial Paralysis: A Case Report

**DOI:** 10.1155/2012/262958

**Published:** 2012-02-09

**Authors:** M. Chihani, A. Aljalil, M. Touati, B. Bouaity, H. Ammar

**Affiliations:** Department of Oto-Rhino-Laryngology, Avicenna Military Hospital, Marrakesh 40000, Morocco

## Abstract

The posttraumatic cholesteatoma is a rare complication of different types of the temporal bone damage. Its diagnosis is often done after several years of evolution, sometimes even at the stage of complications. A case of posttraumatic cholesteatoma is presented that was revealed by a facial nerve paralysis 23 years after a crash of the external auditory canal underlining the importance of the otoscopic and radiological regular monitoring of the patients with a traumatism of the temporal bone.

## 1. Introduction

The cholesteatoma of the middle ear is a chronic otitis described as dangerous because of the evolutionary risks and the potentially serious complications.

 The posttraumatic cholesteatoma is recognized like a rare late complication of various types of the temporal bone damage.

 We report in this study a case of posttraumatic cholesteatoma revealed by a facial paralysis occurring several years after a crash of the external auditory canal.

## 2. Case Report

 A patient, 51 years old, has consulted for a progressive right facial paralysis evolving for two years. In his antecedents there is a war wound caused by a splinter at the right temporomandibular area dating back over 23 years.

 The examination, carried out on a patient in good general state, finds at the inspection signs of a right complete peripheral facial paralysis, with presence in the ipsilateral preauricular area of an old traumatic scar.

 The otoscopy objectifies a cicatricial total stenosis of the right external auditory meatus.

 The instrumental acoumetry is in favour of a right transmission deafness confirmed by the liminary tonal audiometry. The stapedial reflex is absent on the affected side.

 The remainder of ENT and somatic examination is strictly normal.

**Figure 1:**
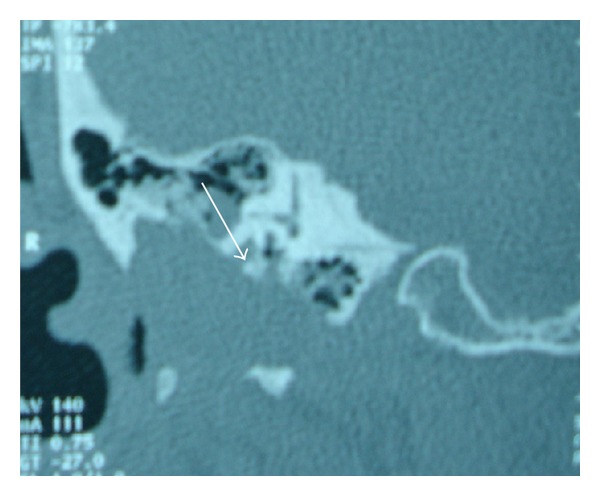
Coronal CT-scan.

**Figure 2:**
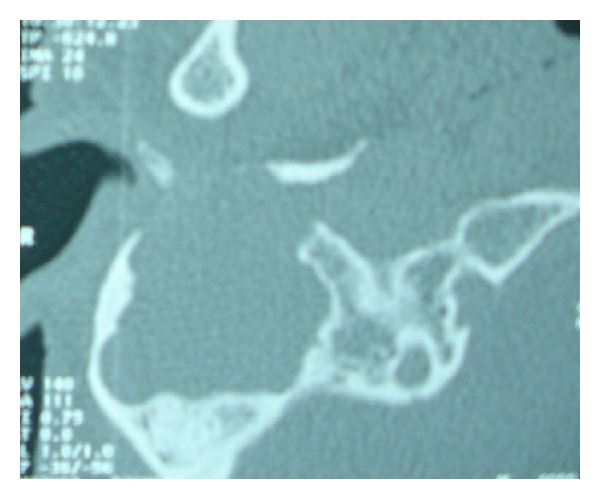
Axial CT-scan.

 A CT-scan of the temporal bone objectifies a crash of the right external auditory canal, quasitotal lysis of the mastoïd, tissue filling the middle ear cavities with total destruction of the ossicular chain, and erosion of 2nd and 3rd portions of right facial nerve canal (Figures 1 and 2).

The surgery confirmed the CT-scan data and the patient benefited from an open technique of tympanoplasty (canal wall down) aimed at eradication of the cholesteatoma and decompression of the facial nerve with repermeabilisation of the external auditory meatus.

 The postoperative courses were simple but no improvement of facial nerve function was noted.

## 3. Discussion

The cholesteatoma is the result of development in the middle ear of a keratinized malpighian epithelium endowed with a potential of desquamation, migration, and erosion [1, 2].

 There are two main types of cholesteatomas: the primitive or congenital cholesteatoma developing behind an intact tympanic membrane, and the acquired cholesteatoma whose origin can be an invasion by progressive colonization of the mucosae after crossing the free edge of a tympanic perforation, an evolution of a retraction pocket, or an iatrogenic or posttraumatic epidermal inclusion by skin incarceration during a fracture involving the external auditory canal [1].

 The posttraumatic cholesteatoma is a rare and often very delayed complication of temporal bone trauma and can remain undetected for years allowing it to develop intensively [3].

 The time interval between the temporal bone damage and the diagnosis of the posttraumatic cholesteatoma is very variable and may range from 1 to 25 years. In the majority of the cases reported in the literature, the interval was more than 10 years [4, 5]. In our case, the latent interval is 23 years.

 In most cases, the evocative clinical signs of a cholesteatoma are fetid otorrhea, otalgy, and hearing loss [1]. In our case, the otorrhea is absent because there is a complete meatal stenosis.

 In the evolved forms, the diagnosis can be revealed by labyrinthian, neuromeningeal, or facial complications, sources of cholesteatoma severity [1].

 The facial paralysis is a rare complication of cholesteatoma with a frequency estimated at 1-2% [1, 6]. Generally, his installation is rapid during an infection but sometimes, it is progressive during an erosion of the facial canal, as in the case of our patient [7].

 The CT-scan currently occupies an essential place in the diagnosis of middle ear cholesteatoma [8, 9] because it can provide semiological arguments in favour of the positive diagnosis with tissue filling the middle ear cavities and signs of osteolysis, specify the extensions, and seek possible complications like a lysis of the tegmen tympani and/or antri, a labyrinthine fistula, or an erosion of the facial nerve canal, what is essential for presurgical planning. The MRI occupies the second place. However, it may provide additional information on the delineation and extension of cholesteatoma and on potential complications [10].

 The therapeutic management is surgical consisting primarily of a canal wall up (closed) or a canal wall down (open) tympanoplasty [11, 12] with treatment of the complications if it is necessary.

## 4. Conclusion

The posttraumatic cholesteatoma is a rare complication of the temporal bone trauma. Its diagnosis is often done in the majority of cases after several years of evolution, sometimes even at the stage of complications that can compromise the functional and even vital prognosis, hence the need for regular monitoring, by otoscopy and/or CT-scan of the temporal bone, of any patient with a temporal bone trauma in order to make an early diagnosis and lead to a therapy as soon as possible.
